# Mesenteric Cysts as Rare Causes of Acute Abdominal Masses: Diagnostic Challenges and Surgical Insights from a Literature Review

**DOI:** 10.3390/jcm14144888

**Published:** 2025-07-10

**Authors:** Laurențiu Augustus Barbu, Nicolae-Dragoș Mărgăritescu, Liliana Cercelaru, Ionică-Daniel Vîlcea, Valeriu Șurlin, Stelian-Stefaniță Mogoantă, Gabriel Florin Răzvan Mogoș, Tiberiu Stefăniță Țenea Cojan, Liviu Vasile

**Affiliations:** 1 Department of Surgery, Railway Clinical Hospital Craiova, University of Medicine and Pharmacy of Craiova, 2 Petru Rares Street, 200349 Craiova, Romania; laurentiu.barbu@umfcv.ro (L.A.B.); gabriel.mogos@umfcv.ro (G.F.R.M.); 2 Department of Surgery, Emergency County Hospital, University of Medicine and Pharmacy of Craiova, 2 Petru Rares Street, 200349 Craiova, Romania; dmargaritescu@yahoo.com (N.-D.M.); vsurlin@gmail.com (V.Ș.); ssmogo@yahoo.com (S.-S.M.); vliviu777@yahoo.com (L.V.); 3 Department of Embryology and Anatomy, University of Medicine and Pharmacy of Craiova, 200349 Craiova, Romania; liliana.cercelaru@umfcv.ro

**Keywords:** mesenteric cyst, laparotomy, complete excision

## Abstract

**Background/Objectives**: Abdominal tumors can trigger acute, life-threatening complications, needing urgent care. Though often slow-growing, they may present suddenly with obstruction, bleeding, or organ compression. This article explores diagnostic challenges in such emergencies and presents a rare case of a giant mesenteric cyst. **Methods**: A PubMed search was conducted to review abdominal tumors in emergencies, focusing on mesenteric cysts. **Results**: A 37-year-old woman with no significant history presented with two weeks of diffuse abdominal pain and distension. Labs showed mild inflammation and low malignancy risk. Imaging revealed a large cystic mass compressing abdominal organs. Surgery found a 35 × 15 cm cyst from the mesenteric root extending into the pelvis and behind the stomach. **Conclusions**: Mesenteric cysts are rare with vague symptoms, needing high suspicion for diagnosis. Imaging helps, but large cysts often require surgery. Complete removal prevents recurrence, and bowel resection may be needed if vital structures are involved. Careful planning, teamwork, and follow-up ensure success.

## 1. Introduction

Abdominal tumors, while often characterized by indolent progression and incidental discovery during routine evaluations, may occasionally present with acute, life-threatening complications. Such emergent manifestations can occur in both benign and malignant lesions and may result from tumor progression, secondary complications, or oncologic therapies. Clinical scenarios involving bowel obstruction, visceral perforation, hemorrhage, tumor lysis, or compressive phenomena require expedited diagnostic workup, advanced imaging, and in many cases, urgent surgical or interventional management coordinated through a multidisciplinary team.

The aim of this article is to analyze the diagnostic challenges associated with mesenteric cysts presenting as acute abdominal masses in emergency settings, with an emphasis on distinguishing them from other benign and malignant lesions. A rare clinical case of a giant mesenteric cyst is presented, highlighting its clinical, imaging, and surgical management features.

This manuscript provides relevant clinical insights for emergency care providers, including surgeons, radiologists, and attending physicians, by addressing an uncommon yet diagnostically challenging abdominal condition that may present as an acute surgical emergency. The early identification of atypical imaging features and the application of a broad differential diagnosis are essential to avoid delayed or inappropriate interventions. The case of a giant mesenteric cyst presented herein illustrates the critical need for interdisciplinary collaboration in emergency decision-making and highlights the importance of awareness regarding rare but treatable causes of abdominal pathology.

### 1.1. General Classification: Benign vs. Malignant

The distinction between benign and malignant abdominal tumors is fundamental to formulating an appropriate therapeutic strategy and estimating patient prognosis. Benign tumors are typically well-demarcated and non-invasive, demonstrating limited growth potential without metastatic behavior. These lesions are generally associated with a favorable clinical course and may be managed conservatively or surgically, depending on their anatomical location, size, and symptomatology. Frequently encountered benign abdominal tumors include uterine fibroids, ovarian cysts, hepatic adenomas, and intestinal adenomatous polyps. While often asymptomatic, such tumors may become clinically significant when they attain substantial dimensions, potentially causing complications such as gastrointestinal obstruction or intratumoral hemorrhage.

Malignant abdominal tumors exhibit uncontrolled cellular proliferation, with a marked capacity for local invasion and distant metastasis. Their aggressive clinical course necessitates prompt diagnosis and a multidisciplinary treatment strategy, often involving surgery, chemotherapy, and radiotherapy. Frequent entities include colorectal carcinoma, pancreatic cancer, hepatocellular carcinoma, and intra-abdominal sarcomas. Due to their rapid progression and potential for acute presentations such as obstruction or hemorrhage, early recognition is critical. Differentiating between benign and malignant lesions remains essential in emergency settings, guiding both prognosis and the urgency of therapeutic interventions.

Frequent abdominal pathologies encountered in emergency settings include ovarian tumors, cystic formations, intra-abdominal abscesses, neoplastic masses, and pancreatic pseudocysts. Ovarian tumors, whether benign or malignant, may manifest acutely with pelvic pain, abdominal distension, or menstrual irregularities. Abscesses secondary to intra-abdominal infections can progress rapidly, presenting with localized tenderness, fever, and risk of sepsis. Neoplastic lesions—primary or metastatic—may cause acute symptoms such as bowel obstruction, hemorrhage, or organ compression. Pancreatic pseudocysts, often post-pancreatitis, can become symptomatic due to mass effects or complications like infection or rupture.

### 1.2. Diagnostic Challenges in Emergency Settings

**Non-specific clinical symptoms**: Abdominal tumors often present with vague symptoms that can resemble other medical issues, complicating early diagnosis.

**Initial imaging tools**: Abdominal ultrasound and CT scans (both native and contrast-enhanced) are key in the initial assessment to determine the tumor’s characteristics, location, and extent.

**Consequences of delayed or incorrect diagnosis**: Delays or misdiagnosis can lead to worse outcomes, such as complications, metastasis, or damage to nearby organs.

**Urgency of intervention**: In some cases, immediate action is required, even before a full diagnosis is made, to prevent the condition from worsening or to stabilize the patient.

Mesenteric cysts are uncommon intra-abdominal tumors, with an incidence of approximately 1 in 250,000 hospital admissions. They are often discovered incidentally during imaging studies or during surgery performed for other abdominal complications [1,2].

The first mesenteric cyst was described by Italian anatomist Benevieni in 1507 during an autopsy of a child, followed by Rokitansky’s identification of a chylous variant in 1842 and Tillaux’s first surgical excision in 1880 [1,3,4]. These cysts are classified into several histological subtypes, including lymphatic (lymphangioma, simple lymphatic cysts), mesothelial (simple mesothelial cysts, benign and malignant cystic mesothelioma), enteric (enteric cysts, enteric duplications), urogenital, mature cystic teratomas (dermoid cysts), and non-pancreatic pseudocysts [3,5].

Mesenteric cysts are rare intra-abdominal lesions of uncertain etiology, thought to arise from mesenteric tissues and potentially located anywhere along the gastrointestinal tract from the duodenum to the rectum. They most frequently occur in the ileal mesentery (67%) and the mesocolon (33%) [1,3]. Although several theories have been proposed—such as trauma, infection, and impaired lymphatic drainage—none have been conclusively validated. These cysts can vary in presentation, appearing as single or multiple, with unilocular or multilocular formations, and their contents may range from serous and purulent to chylous or hemorrhagic fluid. Complete surgical excision remains the first-line treatment, offering excellent prognosis and preventing recurrence. Malignant transformation is rare, reported in only about 3% of cases [1,6,7].

Clinically, mesenteric cysts present with non-specific symptoms, which may include chronic abdominal pain, nausea, vomiting, palpable abdominal masses, bowel obstruction, hemorrhage, or the compression of adjacent organs [3,4,8,9,10]. Additionally, in some cases, the cyst may rupture following abdominal trauma.

## 2. Case Presentation

**Figure 1:**
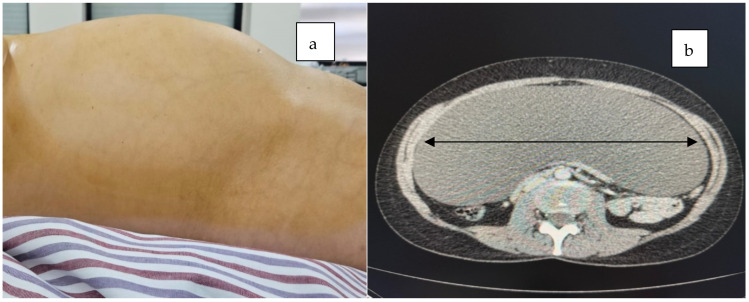
A distended abdomen was observed, predominantly in the middle and lower quadrants (**a**), and the cross-sectional CT scan revealed a large tumor mass (black double arrow) occupying the entire section (**b**).

A 37-year-old female patient was admitted with diffuse abdominal pain persisting for two weeks and with no significant medical history, including no abdominal trauma or prior surgeries. Upon abdominal examination, a distended abdomen was noted, predominantly in the middle and lower quadrants (Figure 1), with bowel sounds present and no signs of peritoneal irritation. Her menstrual cycle was normal, and she reported no vaginal discharge or abnormal bleeding. The patient’s general condition was stable, and her vital signs were within normal limits. A rectal examination revealed no pathological findings.

The patient’s results showed normal hemoglobin, platelet count, and CA-125, a mildly elevated white blood cell count and CA 19-9, normal CEA, and a low ROMA score, suggesting a low risk of malignancy but possible mild inflammation (Table 1).

**Figure 2:**
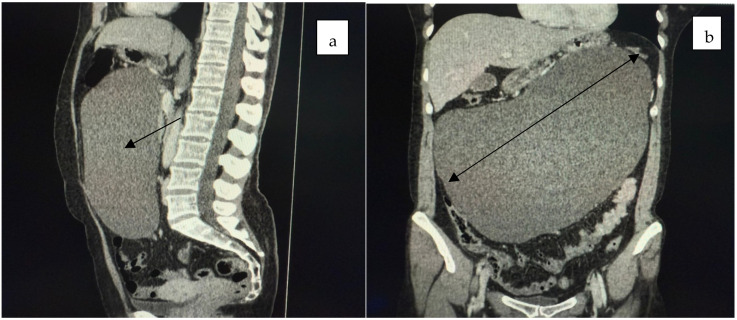
The sagittal ((**a**), black aroow) and axial ((**b**), black double arrow) CT scans of the abdomen and pelvis revealed a large tumor mass occupying the entire abdominal and pelvic cavity, causing partial compression of the stomach and exerting a mass effect on both the large and small intestines.

**Figure 3:**
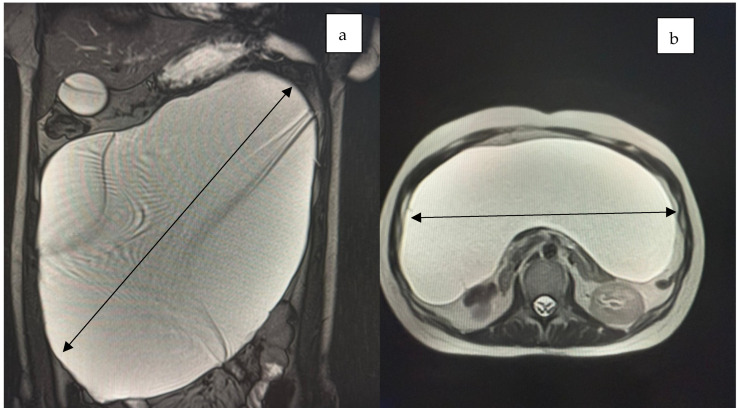
The axial ((**a**), black double arrow) and cross-section ((**b**), black double arrow) MRI scans of the abdomen revealed a tumor mass occupying the entire peritoneal cavity with fluid content.

Computed tomography and MRI revealed a large cystic mass filling the entire abdominal and pelvic cavity, causing partial stomach compression and exerting a mass effect on both the large and small intestines (Figure 1, Figure 2 and Figure 3).

**Figure 4:**
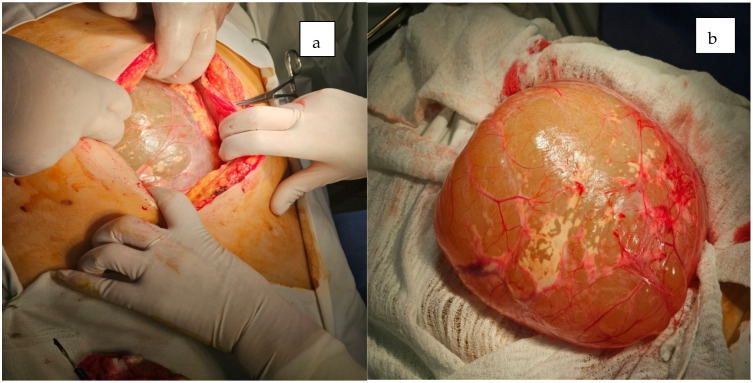
Intraoperative (**a**) and perioperative findings revealed a giant cystic mass upon (**b**) opening the peritoneal cavity during laparotomy.

**Figure 5:**
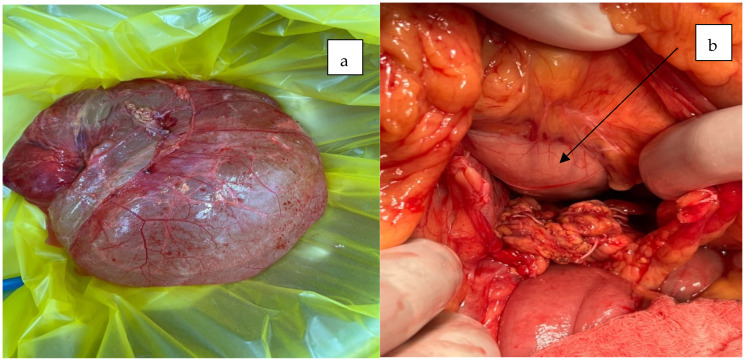
Following complete enucleation of the mesenteric cyst (**a**), intraoperative assessment revealed cranial extension of the cyst into the omental bursa, where it exerted pressure on the posterior gastric wall ((**b**), black arrow).

The patient underwent a median laparotomy, during which a large cystic mass measuring approximately 35 × 15 cm was identified. The mass originated from the root of the mesentery, extended inferiorly to the rectouterine pouch, and superiorly entered the omental bursa posterior to the stomach (Figure 4 and Figure 5).

**Figure 6:**
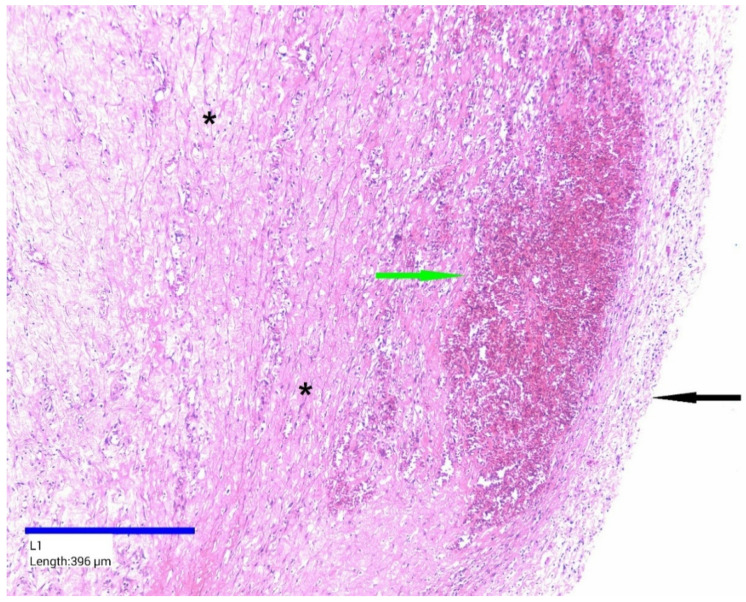
Cystic wall, HE staining; ×100. Epithelial lining absent—black arrow; hemorrhagic foci—green arrow; interstitial fibrosis—black asterisk.

**Figure 7:**
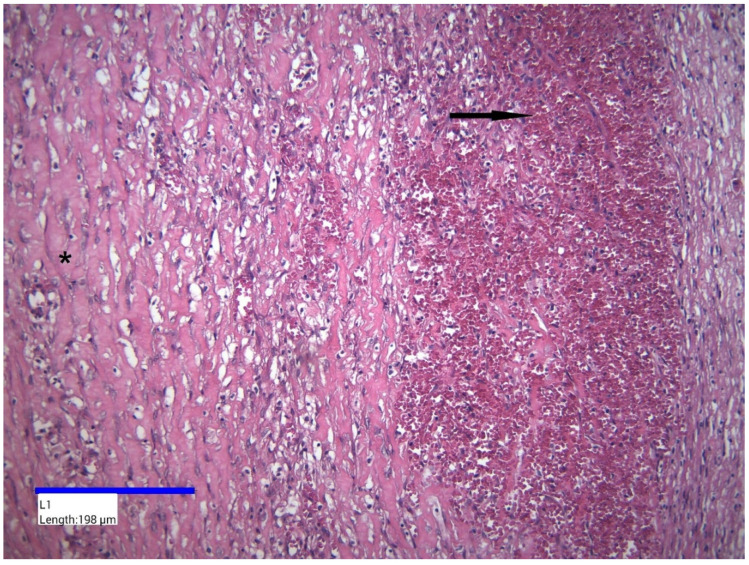
Cystic wall, HE staining; ×200. Hemorrhagic foci—black arrow; interstitial fibrosis—black asterisk.

Histopathological examination revealed a cystic wall of variable thickness, composed predominantly of fibroconnective tissue, with no identifiable epithelial lining (Figure 6). The wall exhibited extensive interstitial fibrosis and a chronic inflammatory infiltrate, primarily consisting of lymphocytes and plasma cells. The intensity of the inflammatory response varied but was generally diffuse in distribution. Numerous small blood vessels displaying vascular hyperemia were also noted (Figure 7). Additionally, multiple intramural foci of hemorrhage were observed.

## 3. Discussion

Abdominal tumors, particularly mesenteric cysts, present considerable diagnostic challenges in emergency settings due to their nonspecific symptoms and diverse clinical manifestations. These tumors often present with vague abdominal pain, distension, and gastrointestinal symptoms such as nausea and vomiting.

Mesenteric cysts are often discovered incidentally, either during routine imaging or surgical procedures or in the context of complications such as bowel obstruction, torsion, or hemorrhage.

In a study conducted by Prakash et al. involving 17 cases, it was found that 82% of patients presented with pain, 61% exhibited a palpable abdominal mass, 45% experienced nausea and vomiting, and 6% had diarrhea [1,11,12]. Mesenteric cysts are commonly diagnosed incidentally through imaging studies or during laparotomy procedures [3]. These cysts, when located in the retroperitoneum, are characterized by a lining composed of endothelial or mesothelial cells, which distinguishes them from mesenteric cysts [1]. Mesenteric cysts are located within the mesentery and are usually of lymphatic or mesothelial origin, whereas retroperitoneal cysts develop behind the peritoneum and typically arise from embryologic remnants of the urogenital or lymphatic systems.

In terms of common locations, a study by Saviano et al. involving 162 patients revealed that 60% of mesenteric cysts were situated in the mesentery of the small intestine, 24% in the mesentery of the large intestine, 14.5% in the retroperitoneum, and in 1.5% of cases, the exact location remained undefined [1,13].

Fewer than 1000 cases have been reported in the literature to date. Approximately 40% of cases are detected incidentally, while others present with vague, non-specific abdominal symptoms. In about 10% of patients, the cysts become clinically apparent due to complications such as intestinal obstruction, volvulus, torsion, or even hemodynamic instability. Diagnosis remains challenging, as both clinical presentation and imaging findings are typically non-specific, often necessitating surgical exploration for definitive identification [14].

Mesenteric cysts are rare abdominal masses, typically originating from the mesentery, and they vary in both size and location. Their clinical presentation is often nonspecific, leading to diagnostic confusion with other abdominal conditions, including ovarian cysts, lymphangiomas, or abscesses. In emergency settings, mesenteric cysts are commonly identified through imaging studies performed for unrelated symptoms.

Radiologic techniques, such as ultrasound, computed tomography (CT), and magnetic resonance imaging (MRI), are essential in diagnosing mesenteric cysts. These imaging modalities help differentiate mesenteric cysts from other abdominal pathologies by revealing well-circumscribed, fluid-filled masses in the mesentery. Additionally, mesenteric cysts may exert pressure on or displace adjacent structures, and their imaging features guide subsequent management decisions.

In the context of smaller cystic retroperitoneal lesions, which are more frequently encountered than the giant cyst presented in our case, the use of endoscopic ultrasound (EUS)-guided fine-needle aspiration or biopsy plays a critical role in the differential diagnosis. EUS allows for precise sampling of the cyst wall or internal components, which can then undergo cytological, histopathological, and immunohistochemical analysis. This approach is particularly useful when imaging findings are inconclusive or when malignancy cannot be excluded. Recent studies underscore the diagnostic value of EUS-guided tissue acquisition, especially when complemented by immunohistochemistry, in differentiating benign cystic lesions from cystic neoplasms or metastatic disease. Moreover, the development of advanced EUS tools has enhanced the ability to obtain high-quality tissue samples, thereby facilitating more accurate and timely diagnoses, which are essential for guiding appropriate therapeutic strategies in emergency and elective settings [15].

The size of mesenteric cysts typically ranges from a few millimeters to several centimeters in diameter; however, some cases present with large cystic masses, which may mimic conditions such as tuberculous ascites in the differential diagnosis [1,2].

There is no definitive pathogenic theory explaining the growth of mesenteric cysts. However, factors such as the inability of the lymphatic system to connect with the venous system or its obstruction due to trauma, infection, or neoplasia are considered important etiological contributors [1,2]. Gross et al. [16] proposed a theory suggesting that mesenteric cysts arise from the benign proliferation of ectopic lymph nodes within the mesentery, independent of the broader lymphatic system, a theory that appears to be widely accepted [1,17].

An alternative hypothesis proposed by Viola et al. suggests that mesenteric cystic lymphangiomas may result from chronic intermittent volvulus [18]. In contrast, Beahrs et al. (1955) classified mesenteric cysts into four types, namely developmental, traumatic, infectious, and neoplastic [19,20]. Notably, mesenteric cystic tumors are unique in their potential to undergo malignancy or recurrence [19,21].

Ros et al. investigated the correlation between histological and imaging classifications of omental and mesenteric cysts, concluding that non-pancreatic cysts may be unilocular or multiloculated, typically located in the mesentery or omentum, exhibiting abundant ultrasound debris and increasing wall thickness on CT. Enteric duplication cysts appear unilocular with an enhancing wall on CT, which serves as a key distinguishing feature for these cystic formations. In contrast, enteric and mesothelial cysts present as anechoic lesions with thin-walled cysts [19,22].

Imaging techniques commonly used for diagnosing mesenteric cysts include ultrasound, MRI, and CT scans [19,23]. MRI offers high sensitivity and specificity, providing detailed information about the cyst’s size [19,24] and its relationship with surrounding structures. CT scans may help identify the cyst’s origin and offer better visualization of calcification in the cystic walls. Ultrasound is useful for assessing the cyst’s contents, the presence of septations, and abdominal fluid accumulation [17,19]. MRI-MRCP is considered the most specific imaging modality for determining the origin, size, and cystic components [19,24].

The initial diagnosis can be challenging because the symptoms of a mesenteric cyst are often vague or nonspecific. For example, abdominal pain or bloating may be mistaken for more common conditions. If the cyst was discovered incidentally or after several investigations, the diagnosis could remain uncertain until confirmed through imaging. Additionally, multiple imaging techniques, such as MRI or CT scans, may be necessary to differentiate the mesenteric cyst from other abdominal masses, which adds to the complexity of establishing a clear diagnosis.

French surgeon Paul Jules Tillaux described a set of clinical signs suggestive of a mesenteric cyst, including a peri-umbilical, freely mobile mass that moves perpendicular to the axis of mesenteric attachment. On percussion, a tympanic sound is noted anteriorly due to overlying bowel loops, while a dull sound posteriorly indicates the fluid-filled nature of the cyst [25].

Behzad et al. reported that a large mesenteric cyst causing small bowel malrotation and the whirlpool sign can lead to mesenteric ischemia. Early recognition is crucial, as such cysts may induce bowel obstruction requiring urgent surgery [26,27].

Mesenteric cystovarian implant syndrome shares features with ovarian remnant syndrome, including a history of oophorectomy or salpingo-oophorectomy (typically bilateral), an inadvertent retention of ovarian tissue, associated pelvic pain or dyspareunia, and symptom resolution following excision of the residual tissue. A review of the literature identified only five reported cases of mesenteric cysts associated with ovarian implant syndrome. Most patients had a history of multiple pelvic surgeries, particularly involving ovarian cyst excision, which may increase the risk of ectopic ovarian implantation. Nonetheless, the rarity of this condition suggests that normal pelvic tissues are generally unfavorable for ovarian implant survival [14].

Given that it is a giant cyst, the large dimensions can complicate both the diagnostic process and the therapeutic approach. A cyst of this size, occupying the entire peritoneal cavity, can be difficult to fully assess, and planning a surgical intervention may become risky due to the challenges posed by its size. It can be extremely difficult to remove it completely without causing damage to surrounding structures.

This large cyst can compress the surrounding organs, including the small or large intestine, liver, kidneys, or blood vessels. This compression can lead to intestinal obstructions or impaired blood circulation in certain areas, which increases the risk of ischemia or other postoperative complications. Furthermore, surgical interventions in these cases can be very delicate, and the medical team must be prepared for a very careful approach to avoid damaging vital organs.

The mesenteric cyst’s close proximity to the stomach caused nonspecific gastrointestinal symptoms, often mimicking giant gastric GISTs. While imaging may be similar, GISTs usually exhibit solid enhancing components, unlike the fluid-filled mesenteric cyst. Definitive diagnosis depends on histopathology [28,29].

A short review of published cases over the past decade identified mesenteric cysts exceeding 8 cm in diameter, frequently associated with nonspecific gastrointestinal symptoms, predominantly abdominal pain and distension (Table 2).

**Table 2:** Literature review of >8 cm mesenteric cyst—symptoms and size.

Author (Ref)	Age at Diagnosis	Sex	Gastrointestinal Symptoms	Cystic Size
**Bolivar-Rodriguez** [30]	49 years	Male	Abdominal pain and abdominal distension	13 × 10 cm
**Ghritlaharey** [31]	8 years	Male	Pain, vomiting, abdominal distension	10 × 8 cm
**Park** [32]	72 years	Male	No symptoms	13.5 × 9 cm
**Pozzi** [33]	29 years	Male	Episodes of colicky abdominal pain and subjective discomfort	8.5 × 7 cm
**Mickovic** [9]	55 years	Male	Mild abdominal pain	15 × 12 cm
**Al Booq** [34]	42 years	Male	Abdominal pain	14 × 10 cm
**Pithawa** [1]	7 years	Male	Abdominal pain	11 × 7 cm
**Dioscoridi** [35]	61 years	Female	Asymptomatic	10 cm
**Li** [36]	25 years	Male	Abdominal pain and weight loss	15 × 4 cm
**Yoldemir** [37]	48 years	Female	Abdominal discomfort and nausea	22 × 10 cm
**Lee** [38]	34 years	Female	Abdominal discomfort and distension	10 × 10 cm
**Blanco** [39]	25 years	Male	Abdominal pain	10 × 15 cm
**Reddy** [40]	22 years	Female	Abdominal pain	10 × 10 cm
**Yagmur** [41]	20 years	Female	Distended abdomen	33 × 14 cm
**Karhan** [42]	5 years	Male	Abdominal distension	25 × 20 cm
**Rosado** [43]	55 years	Male	No symptoms	15 × 10 cm

### 3.1. Diagnostic Challenges

The initial diagnosis in this case was uncertain due to several factors. The patient presented with nonspecific symptoms, including diffuse abdominal pain, progressive distension, and postprandial discomfort, without clinical signs of bowel obstruction or acute inflammation. Upon physical examination, the abdominal mass was difficult to delineate, raising multiple diagnostic possibilities—ranging from retroperitoneal or ovarian masses to malignant neoplasms [1,44].

Initial emergency ultrasonography revealed a large, cystic abdominal lesion but was unable to accurately determine its origin. Although contrast-enhanced computed tomography (CT) provided further anatomical detail, the differentiation between a mesenteric cyst, lymphangioma, and pancreatic pseudocyst remained difficult due to the lesion’s substantial size and the compression of adjacent structures [22].

The tumor’s diameter exceeded 15 cm, occupying a large portion of the mid and lower abdomen. It displaced and compressed intestinal loops and mesenteric vessels, complicating the preoperative surgical strategy and increasing the risk of intraoperative complications [22].

Although mesenteric cysts are typically benign entities, the differential diagnosis of cystic or hemorrhagic intra-abdominal lesions in emergency settings must also account for aggressive malignancies such as retroperitoneal sarcomas. Samà et al. reported a rare case of a spontaneously ruptured retroperitoneal leiomyosarcoma complicated by hemoperitoneum, necessitating urgent surgical management to avert fatal progression [45]. This case illustrates the critical importance of maintaining a high index of suspicion for malignant processes, particularly in acute presentations characterized by nonspecific symptoms and rapid clinical deterioration. Preoperative rupture and hemorrhagic dissemination not only complicate immediate management but are also associated with significantly worse oncologic outcomes, including higher rates of local recurrence and decreased overall survival. Accordingly, emergent presentations of large abdominal masses require a judicious and multidisciplinary approach that integrates imaging, surgical planning, and oncologic expertise to optimize both diagnostic accuracy and therapeutic outcomes.

### 3.2. Intraoperative Challenges

Surgical exploration confirmed a giant mesenteric cyst. Key intraoperative challenges included the following:

The meticulous dissection of the cyst from the mesenteric root without compromising mesenteric vascularization;

The prevention of cyst rupture, which could lead to contamination of the surgical field;

The identification of the correct cleavage plane to allow for safe and complete enucleation of the mass.

Given the size and location of the lesion, the risk of vascular injury or intestinal ischemia was significant and required constant intraoperative reassessment.

### 3.3. Clinical and Surgical Lessons

This case emphasizes the importance of an open diagnostic mindset when encountering atypical abdominal masses in an emergency setting. Clinicians should include rare entities such as mesenteric cysts in the differential diagnosis, especially when imaging reveals a well-defined, fluid-filled mass without signs of solid components [46].

Furthermore, the case highlights the strengths and limitations of diagnostic imaging in the context of distorted anatomy. While ultrasonography and CT are valuable, they may not always definitively identify the lesion’s origin, particularly in cases involving large, space-occupying masses. In such scenarios, surgical exploration becomes both diagnostic and therapeutic [47].

From a surgical perspective, the key takeaways are as follows:

The necessity of thorough preoperative planning;

A detailed understanding of mesenteric vascular anatomy;

The implementation of refined surgical techniques to minimize the risk of hemorrhage, intestinal ischemia, or intra-abdominal contamination.

Finally, this case underscores the need for emergency surgeons to make prompt, well-reasoned decisions under pressure, often with limited preoperative data—a hallmark of acute surgical care.

In the case of a mesenteric cyst, both the clinician and the emergency surgeon can learn several valuable lessons regarding diagnosis and treatment.

1. Differential Diagnosis and Use of Advanced Imaging

It is crucial to perform a proper differential diagnosis, as the symptoms of a mesenteric cyst can be vague or mimic other more common abdominal conditions (such as appendicitis, intestinal obstructions, ovarian tumors, or other abdominal masses).

Advanced imaging techniques like MRI and CT scans are essential for confirming the diagnosis and differentiating a mesenteric cyst from other pathologies. These images allow for the assessment of the cyst’s size, location, and its relationship to neighboring organs.

2. Assessment of Size and Impact on Adjacent Organs

Large mesenteric cysts occupying the entire peritoneal cavity can compress surrounding organs and affect their function. The clinician must be aware of potential complications such as intestinal obstructions or the compression of major vessels, which could lead to ischemia.

Understanding the impact on neighboring organs is essential for planning appropriate treatment and preventing intra- and postoperative complications.

3. Surgical Management

Surgical interventions for large mesenteric cysts are complex and require careful consideration. The emergency surgeon must be prepared for the challenges encountered during surgery, such as the risk of damaging vital organs (intestine, liver, blood vessels).

It is important to assess anesthetic risks and decide on the best surgical technique. Surgeons can also learn the importance of a multidisciplinary team, including radiologists, anesthesiologists, and other specialists, to ensure optimal case management.

4. Postoperative Monitoring and Management of Complications

After surgery, the clinician must be prepared to closely monitor the patient for potential complications such as infections, hemorrhages, or recurrences.

Long-term follow-up is also crucial to detect any changes in the cyst or the patient’s health status.

5. Importance of Early Diagnosis

The early identification and treatment of a mesenteric cyst can reduce the risks associated with serious complications such as the perforation, necrosis, or compression of vital organs. This highlights the need for a rapid and accurate diagnosis, as well as timely intervention.

Thus, both the clinician and the emergency surgeon can learn the importance of thorough assessment, the appropriate use of imaging technologies, careful surgical planning, and continuous postoperative monitoring in managing such a complex case.

#### Practical Recommendations for Future Similar Emergency Cases—Giant Mesenteric Cyst

1. Maintain a High Index of Clinical Suspicion

In patients with significant abdominal distension, vague pain, or signs of intra-abdominal compression, rare diagnoses like a mesenteric cyst should be considered, especially when common causes are excluded.

2. Prompt and Comprehensive Imaging

An urgent abdomino-pelvic CT scan is recommended, followed by MRI if the diagnosis remains unclear. Imaging should carefully evaluate the cyst’s relationship with major vessels and adjacent organs to avoid intraoperative surprises.

3. Early Surgical Consultation

Even in clinically stable cases, the early involvement of a surgical team is essential to assess the feasibility of surgery and to plan the operative strategy.

4. Careful Surgical Planning

If the patient is stable, preoperative optimization is advised, and the procedure should be carried out by an experienced surgical team.

Surgeons should anticipate the need for extensive dissection, potential bowel resection, or even vascular control if the cyst is adherent to major structures.

5. Multidisciplinary Approach

Collaboration between the surgeon, anesthesiologist, radiologist, and if necessary, a vascular surgeon or gynecologist is essential for optimal case management.

6. Close Postoperative Monitoring and Follow-up

Patients should be monitored for potential complications such as bleeding, infection, postoperative ileus, or recurrence.

Follow-up imaging (CT or MRI) at 3–6 months postoperatively is recommended, especially in cases where complete excision was not possible.

7. Case Documentation and Reporting

Given the rarity of this condition, documenting the case can help improve medical knowledge. Clinicians are encouraged to prepare a case report for publication or presentation in academic settings.

Definitive surgical treatment includes complete enucleation or cystectomy, which can be performed through laparotomy or laparoscopy. In cases where the cyst is closely adherent to the small intestine or to vascular structures supplying that segment, enterectomy may be necessary. Recurrence is rare, and the condition is associated with an excellent prognosis [1,17].

The definitive management of mesenteric cysts is surgical, with the primary goal being complete excision to minimize the risk of recurrence and complications. Two main surgical approaches are commonly employed, namely complete enucleation and cystectomy.

Complete enucleation is often feasible in cases where the cyst is well-demarcated and not infiltrating adjacent tissues. This method allows for precise dissection and removal without the need for resecting surrounding structures. Alternatively, cystectomy, involving removal of the cyst along with a portion of the mesentery, may be indicated when enucleation is not technically possible. Both procedures can be performed via laparotomy or laparoscopy, with minimally invasive techniques increasingly favored due to their association with shorter hospital stays, reduced postoperative pain, and faster recovery.

In cases where the cyst is intimately associated with the small intestine or lies in proximity to major mesenteric vessels, complete removal may necessitate segmental bowel resection (enterectomy) to avoid ischemia or compromising intestinal viability. Such situations require careful preoperative imaging and intraoperative assessment to guide surgical decision-making.

Regardless of the chosen technique, the key principle remains total removal of the cyst wall, as incomplete excision is associated with a higher risk of recurrence. However, when complete excision is achieved, the prognosis is typically excellent and recurrence is rare.

## 4. Conclusions

Mesenteric cysts are rare and often present with vague symptoms, requiring high clinical suspicion for diagnosis, especially in emergencies. Imaging aids diagnosis, but large or complex cysts often require surgical exploration for confirmation and treatment. Surgery is the main treatment, with full cyst removal key to avoiding recurrence. Bowel resection may be needed if vital structures are affected. Teamwork and careful surgery are vital for giant mesenteric cysts. Success relies on planning, precision, and close follow-up.

## Figures and Tables

**Table 1:** Laboratory tests upon admission.

Parameter	Laboratory Results
**White blood cell**	11 × 10^3^ cells/µL
**Hemoglobin**	13.2 g/dL
**Platelet count**	200 × 10^3^ cells/µL
**Carbohydrate antigen 19.9**	60.9 U/L
**Carcioembryonic antigen**	1.39 ng/mL
**Carbohydrate antigen 125**	23.6 U/mL
**ROMA score**	4.4%

## Data Availability

The data presented in this study are available upon request from the corresponding author. The data are not publicly available due to patient confidentiality.

## References

[1] Pithawa A.K., Bansal A.S., Kochar S.P. (2014). Mesenteric cyst: A rare intra-abdominal tumour. Med. J. Armed Forces India.

[2] Miliaras S., Trygonis S., Papandoniou A., Kalamaras S., Trygonis C., Kiskinis D. (2006). Mesenteric cyst of the descending colon: Report of a case. Acta Chir. Belg..

[3] Suldrup F., Uad P., Vaccaro A., Mazza M., Santino J., Mazza O. (2024). Mesenteric root pseudocyst: Finding in an asymptomatic patient-a case report. Surg. Case Rep..

[4] Mohanty S.K., Bal R.K., Maudar K.K. (1998). Mesenteric cyst—An unusual presentation. J. Pediatr. Surg..

[5] Liew S.C., Glenn D.C., Storey D.W. (1994). Mesenteric cyst. Aust. N. Z. J. Surg..

[6] Xiao Y., Chaudhari S., Khattak T., Tiesenga F. (2022). A Rare Case of Abdominal Tumor: Mesenteric Cyst. Cureus.

[7] Kurtz R.J., Heimann T.M., Holt J., Beck A.R. (1986). Mesenteric and retroperitoneal cysts. Ann. Surg..

[8] Kaushik K., Pratap A., Naik B., Datta Sai Subramanyam A., Ansari M.A. (2023). Intact Excision of a Mesenteric Pseudocyst. Cureus..

[9] Micković S., Bezmarević M., Nikolić-Micković I., Mitrović M., Tufegdzić I., Mirković D., Sekulović L., Trifunović B. (2014). Traumatic mesenteric pseudocyst. Vojn. Pregl..

[10] Milijkovic D., Gmijovic D., Radojkovic M., Gligorijevic J., Radovanovic Z. (2007). Mesenteric cyst. Arch. Oncol..

[11] Raghupati R.K., Krishnamurthy P., Rajamani J., Babuji N., Diriviraj R., Mohan N.V., Swamy R.N., Gurunathan S., Natarajan M. (2003). Intraabdominal cystic swelling in children e laparoscopic approach, our experience. J. Indian. Paediatr. Surg..

[12] Prakash A., Agrawal A., Gupta R.K., Sanghvi B., Parelkar S. (2010). Early management of mesenteric cyst prevents catastrophes: A single centre analysis of 17 cases. Afr. J. Paediatr. Surg..

[13] Saviano M.S., Fundarò S., Gelmini R., Begossi G., Perrone S., Farinetti A., Criscuolo M. (1999). Mesenteric cystic neoformations: Report of two cases. Surg. Today.

[14] El-Agwany A.M.S. (2016). Huge mesenteric cyst: Pelvic cysts differential diagnosis dilemma. Egypt. J. Radiol. Nucl. Med..

[15] Crinò S.F., Bernardoni L., Manfrin E., Parisi A., Gabbrielli A. (2016). Endoscopic ultrasound features of pancreatic schwannoma. Endosc. Ultrasound..

[16] Gross R.E. (1953). The Surgery of Infancy and Childhood: Its Principles and Techniques.

[17] Mason J.E., Soper N.J., Brunt L.M. (2001). Laparoscopic excision of mesenteric cysts: A report of two cases. Surg. Laparosc. Endosc. Percutan Tech..

[18] Weeda V.B., Booij K.A., Aronson D.C. (2008). Mesenteric cystic lymphangioma: A congenital and an acquired anomaly? Two cases and a review of the literature. J. Pediatr. Surg..

[19] Guraya S.Y., Salman S., Almaramhy H.H. (2011). Giant mesenteric cyst. Clin. Pract..

[20] Beahrs O.H., Judd E.S., Dockerty M.B. (1950). Chylous cysts of the abdomen. Surg. Clin. N. Am..

[21] Mennemeyer R., Smith M. (1979). Multicystic, peritoneal mesothelioma: A report with electron microscopy of a case mimicking intra-abdominal cystic hygroma (lymphangioma). Cancer.

[22] Ros P.R., Olmsted W.W., Moser R.P., Dachman A.H., Hjermstad B.H., Sobin L.H. (1987). Mesenteric and omental cysts: Histologic classification with imaging correlation. Radiology.

[23] Falidas E., Mathioulakis S., Vlachos K., Pavlakis E., Anyfantakis G., Villias C. (2011). Traumatic mesenteric cyst after blunt abdominal trauma. Int. J. Surg. Case Rep..

[24] Shamiyeh A., Rieger R., Schrenk P., Wayand W. (1999). Role of laparoscopic surgery in treatment of mesenteric cysts. Surg. Endosc..

[25] Mzee S.A.S., Ali M.M.M., Mutisya C., Shaibu Z., Mutai A.K., Al-Busaidy S.S.S., Danbala I.A. (2024). Mesenteric cyst manifested as partial intestinal obstruction. Clin. Med. Rev. Case Rep..

[26] Azimi B., Bagherian Lemraski S., Kouchak Hosseini S.P., Khoshnoudi H., Aghaei M., Haghbin Toutounchi A. (2023). Small bowel volvulus and mesenteric ischemia induced by mesenteric cystic lymphangioma in an adult and literature review: A case report. Int. J. Surg. Case Rep..

[27] Barbu L.A., Mărgăritescu N.D., Cercelaru L., Caragea D.C., Vîlcea I.D., Șurlin V., Mogoantă S.Ș., Mogoș G.F.R., Vasile L., Țenea Cojan T.Ș. (2025). Can Thrombosed Abdominal Aortic Dissecting Aneurysm Cause Mesenteric Artery Thrombosis and Ischemic Colitis?-A Case Report and a Review of Literature. J. Clin. Med..

[28] Miettinen M., Lasota J. (2006). Gastrointestinal stromal tumors: Pathology and prognosis at different sites. Semin. Diagn. Pathol..

[29] Barbu L.A., Mărgăritescu N.D., Ghiluşi M.C., Belivacă D., Georgescu E.F., Ghelase Ş.M., Marinescu D. (2016). Severe upper gastrointestinal bleeding from gastrointestinal stromal tumor of the stomach. Rom. J. Morphol. Embryol..

[30] Bolívar-Rodríguez M.A., Cazarez-Aguilar M.A., Luna-Madrid E.E., Morgan-Ortiz F. (2015). Infected jejunal mesenteric pseudocyst: A case report. Cirugía Y Cirujanos.

[31] Ghritlaharey R.K., More S. (2014). Chylolymphatic Cyst of Mesentery of Terminal Ileum: A Case Report in 8 Year-old Boy. J. Clin. Diagn. Res..

[32] Park S.E., Jeon T.J., Park J.Y. (2014). Mesenteric pseudocyst of the transverse colon: Unusual presentation of more common pathology. BMJ Case Rep..

[33] Pozzi G., Ferrarese A., Borello A., Catalano S., Surace A., Marola S., Gentile V., Martino V., Solej M., Nano M. (2014). Percutaneous drainage and sclerosis of mesenteric cysts: Literature overview and report of an innovative approach. Int. J. Surg..

[34] Al Booq Y., Hussain S.S., Elmy M. (2014). Giant chylolymphatic mesenteric cyst and its successful enucleation: A case report. Int. J. Surg. Case Rep..

[35] Dioscoridi L., Perri G., Freschi G. (2014). Chylous mesenteric cysts: A rare surgical challenge. J. Surg. Case Rep..

[36] Li B.L., Huang X., Zheng C.J., Zhou J.L., Zhao Y.P. (2013). Ileal duplication mimicking intestinal intussusception: A congenital condition rarely reported in adult. World J. Gastroenterol..

[37] Yoldemir T., Erenus M. (2013). Fatty necrosis of a mesenteric cyst in a woman initially diagnosed with a large ovarian cystic mass. J. Obs. Gynaecol..

[38] Lee D.L., Madhuvrata P., Reed M.W., Balasubramanian S.P. (2016). Chylous mesenteric cyst: A diagnostic dilemma. Asian J. Surg..

[39] Blanco A., Sonntag C., Giese A. (2013). Right lower quadrant abdominal pain—The usual suspects? Diagnosis and therapy of a symptomatic mesenteric cyst. Dtsch. Med. Wochenschr..

[40] Reddy G.R., Gunadal S., Banda V.R., Banda N.R. (2013). Infected mesenteric cyst. BMJ Case Rep..

[41] Yagmur Y., Akbulut S., Gumus S., Babur M., Can M.A. (2016). Case Report of Four Different Primary Mesenteric Neoplasms and Review of Literature. Iran. Red. Crescent Med. J..

[42] Karhan A.N., Soyer T., Gunes A., Talim B., Karnak I., Oguz B., Saltik Temizel I.N. (2016). Giant Omental Cyst (Lymphangioma) Mimicking Ascites and Tuberculosis. Iran. J. Radiol..

[43] Rosado C.R.B., Machado D.S., de Magalhães Esteves J., Moreira R.F.D.S., de Castro Neves C.M. (2016). A Case of Mesenteric Pseudocyst Causing Massive Abdominal Swelling. Eur. J. Case Rep. Intern. Med..

[44] Barbu L.A., Vasile L., Țenea-Cojan T.Ș., Mogoș G.F.R., Șurlin V., Vîlcea I.D., Cercelaru L., Mogoantă S.Ș., Mărgăritescu N.D. (2025). Endometrioid adenofibroma of ovary—A literature review. Rom. J. Morphol. Embryol..

[45] Samà L., Tzanis D., Bouhadiba T., Bonvalot S. (2021). Emergency Retroperitoneal Sarcoma Surgery for Preoperative Rupture and Hemoperitoneum: A Case Report. Cureus.

[46] de Perrot M., Bründler M.A., Tötsch M., Mentha G., Morel P. (2000). Mesenteric cysts: Toward less confusion? A review. Surgery.

[47] Losanoff J.E., Kjossev K.T., El-Sherif A., Richman B.W., Weaver D.W. (2003). Mesenteric cysts. J. Am. Coll. Surg..

